# ACSL1 Inhibits ALV-J Replication by IFN-Ⅰ Signaling and PI3K/Akt Pathway

**DOI:** 10.3389/fimmu.2021.774323

**Published:** 2021-10-29

**Authors:** Qihong Zhang, Tingting Xie, Guodong Mo, Zihao Zhang, Ling Lin, Xiquan Zhang

**Affiliations:** ^1^ Guangdong Provincial Key Laboratory of Agro-Animal Genomics and Molecular Breeding, College of Animal Science, South China Agricultural University, Guangzhou, China; ^2^ Key Lab of Chicken Genetics, Breeding and Reproduction, Ministry of Agriculture, Guangzhou, China; ^3^ State Key Laboratory for Conservation and Utilization of Subtropical Agro-Bioresources, South China Agricultural University, Guangzhou, China

**Keywords:** ACSL1, ALV-J, IFN-Ⅰ, PI3K/Akt, apoptosis

## Abstract

J subgroup avian leukosis virus (ALV-J) infection causes serious immunosuppression problems, leading to hematopoietic malignancy tumors in chicken. It has been demonstrated that interferon-stimulated genes (ISGs) could limit ALV-J replication; nevertheless, the underlying mechanisms remain obscure. Here, we demonstrate that Long-chain Acyl-CoA synthetase 1 (ACSL1) is an interferon (IFN)-stimulated gene that specifically restricts the replication of ALV-J due to the higher IFN-I production. More importantly, ACSL1 induces primary monocyte-derived macrophages (MDMs) to pro-inflammatory phenotypic states during ALV-J infection, and ACSL1 mediates apoptosis through the PI3K/Akt signaling pathway in ALV-J-infected primary monocyte-derived macrophages (MDMs). Overall, these results provide evidence that ACSL1 contributes to the antiviral response against ALV-J.

## Introduction

J subgroup avian leukosis virus (ALV-J) is an alpharetro virus that mainly induces hematopoietic malignancy with myeloid leukemia in chicken (1, 2). ALV-J infection is closely related to the onset of visceral and vascular neoplasms with associated adverse consequences, including stunted growth, decreased egg production, and increased mortality, causing significant economic loss for the poultry industry worldwide (3, 4). Unfortunately, no vaccines or drugs have been exploited to treat the ALV-J infection-triggered disease effectively (5).

The innate immune system is the first line of host defense against pathogen invasion (6). One of the most potent events is the activation of interferon (IFN) pathways (7). When IFNs bind to their receptors, a cascade of signals gets activated, resulting in the activation of several IFN-stimulated genes (ISGs). Indeed, the ISGs play crucial roles and exert diverse functions at multiple levels of antiviral immunity. For instance, the interferon-induced transmembrane (IFITM) family blocks virus replication by inhibiting the virus and host cell membrane fusion (8), the zinc-finger antiviral protein (ZAP) binds to the target RNA to block the translation of viral mRNA (9), and the IFN-induced protein with tetratricopeptide repeats 5 (IFIT5) inhibits RNA virus replication by preventing virus translation and initiating innate immunity (10, 11). Notably, in our previous study, we proved that the ALV-J can be particularly sensitive to IFN-I (12). Moreover, previous studies have demonstrated that overexpression of the immune response gene 1 (IRG1), 2,5-oligoadenylate synthetase (2,5-OAS), and cholesterol-25-hydroxylase (CH25H) could significantly decrease ALV-J replication *in vitro* (12, 13). Despite lots of ISGs have been identified in chicken, the functions of the majority of individual ISGs and precisely how they inhibit replication of ALV-J remain unclear.

Long-chain Acyl-CoA synthetase 1 (ACSL1), a member of the ACSLs family, is responsible for the activation of the most abundant long-chain fatty acids (12–22 carbons) (14, 15). The lipid metabolism network is extensive and intertwined, involving various metabolic functions such as energy production, temperature regulation, and molecular signal synthesis (16, 17). However, deregulated fatty acid metabolism favoring excess lipid biosynthesis and deposition eventually predispose the disease occurrence, such as cardiovascular diseases, metabolic diseases, and cancer (18). Increasing studies support that ACSL1 is deregulated in clinical cancer. For example, ACSL1 is reported to be upregulated in colorectal (19), breast cancer (20), and myeloma (21), while downregulation is found in liver, non-small-cell lung, and blood cancer (19). Interestingly, ACSL1 is induced by Gram-negative bacteria, lipopolysaccharide (LPS) and IFN-γ, and tumor necrosis factor-α (TNF-α) (22). Thus, ACSL1 may have an important role in cancer and the innate immune response. Notably, we have analyzed previously published *in vitro* transcription-sequencing data of ALV-J and IFN-α cotreatment chicken peripheral blood mononuclear cells (PBMCs) and identified ACSL1 as an ISG (12). Thus, we speculate that ACSL1 could be an important ISG to influence ALV-J replication. However, the role and potential mechanism of ACSL1in ALV-J replication is still unknown.

Therefore, in the present study, we investigate the role of ACSL1 in ALV-J replication. Through exogenous expression and small interfering RNA (siRNA) knockdown, we find that ACSL1 inhibits ALV-J replication by enhancing IFN-I production. Further studies demonstrate that ACSL1 induces apoptosis *via* the PI3K/Akt signaling pathway and promotes inflammation in ALV-J-infected primary monocyte-derived macrophages (MDMs). Our results not only reveal the mechanism by which ACSL1 inhibits ALV-J replication, but also extend understanding of the host’s innate immunity to ALV-J infection.

## Materials and Methods

### Reagents and Antibodies

IFN-α/β, IL-1β, and IL-18 ELISA kits were purchased from Shanghai mlbio. Caspase-1, caspase-3, caspase-8 activity assay kits, Total Nitric Oxide (NO) assay kit, Enhanced ATP assay kit, Enhanced mitochondrial membrane potential assay kit, and PI3K inhibitor (LY294002) were purchased from Beyotime Institute of Biotechnology. Recombinant chicken IFN-α was purchased from Cloud clone crop (CCC, USA). Lipofectamine 3000 was purchased from Invitrogen. The following antibodies were used for immunoblot analysis: anti-ALV-J envelope protein JE9 (kindly provided by Dr. Aijian Qin, Yangzhou University, Yangzhou, China), anti-Flag (AF5051, Beyotime), FITC-labeled goat anti-rabbit IgG (H+L) (A0562, Beyotime), anti-STAT1 (70R-51641, Fitzgerald), anti-phosphorylated STAT1 (15H13L67, Invitrogen), anti-Akt (10176-2-AP, Proteintech), anti-phosphorylated Akt (D9E, Cell Signaling), anti-mTOR (bs-1992R, Bioss), anti-phosphorylated mTOR (D9C2, Cell Signaling), anti-IKKα/β (bs-10123R, Bioss), anti-phosphorylated IKK (bs-3229R, Bioss), anti-β-actin (AF5003, Beyotime), Goat anti-rabbit IgG/HRP (bs-0295G, Bioworld), Goat anti-mouse IgG/HRP (bs-0296G, Bioworld).

### Animals, Virus, and Cells

A total of specific-pathogen-free (SPF) White Leghorn chickens were purchased from Guangdong DaHuaNong Animal Health Products Co., Ltd (Guangzhou, China) and housed under pathogen-free conditions. ALV-J strain SCAU-HN06 was kindly provided by Prof. Weisheng Cao, South China Agricultural University. A 10^4^ TCID_50_/0.1 ml of ALV-J SCAU-HN06 were used in this study. DF-1 cells were purchased from the American Type Culture Collection. Chicken primary MDMs were cultured and identified according to previous studies (23, 24).

### Cell Culture and Virus Infection

DF-1 cells were cultured in DMEM (Catalogue number:11995065, Gibco), supplemented with 10% FBS, and maintained at 37°C and 5% CO2. MDMs were cultured in RPMI-1640 medium with 15% chicken serum, 100 U/ml penicillin, and 100 mg/ml streptomycin and maintained at 37°C and 5% CO_2_. For the cells being infected with virus, briefly, DF-1 cells or MDMs were infected with ALV-J strain SCAU-HN06 (10^4^ TCID_50_/0.1 ml) in without DMEM for 2 h, washed by 1×PBS, and incubated with fresh complete medium for the times shown in the figures.

### Plasmids and siRNA

The *ACSL1*, *STAT1*, and *IRF1* genes were cloned into the expression vector pcDNA3.1 with an N-terminal ATG star codon and a C-terminal Flag tag. Lipofectamine 3000 was used for the transfection of expression vector or siRNAs into cells according to the manufacturer’s instructions. The sequences of siRNAs with higher silencing efficiency are as follows:

Chicken ACSL1-siRNA: 5’- CCUCACGACCUACUGGUAUTT dTdT-3’.

Negative control-siRNA: 5’- UUCUCCGAACGUGUCACGUTT dTdT-3’.

These RNA oligos were synthesized at Suzhou GenePharma Co., Ltd.

### RNA Isolation, qRT-PCR, and ELISA

At the indicated time points per experiment, total RNA was extracted using TRIzol reagent (Thermo Fisher) and reverse-transcribed using the PrimeScript RT Reagent kit (Takara) according to the manufacturer’s instructions. The CFX384 instrument (Bio-Rad) with SYBR green fast mixture (Bio-Rad) was used for quantitative real-time PCR analysis. Expression levels were quantified using the 2^−ΔΔCt^ method and normalized to GAPDH. The primer sequences are listed in **Supplementary Table 1**. The concentrations of IFN-α, IFN-β, IL-1β, and IL-18 in culture supernatants were measured using kits from R&D Systems, according to the manufacturer’s instructions.

### Detection of NO, ATP, Mitochondrial Membrane Potential JC1, and Mitochondrial Respiratory Chain Enzymes

Total Nitric Oxide assay kit (Beyotime Biotechnology, Shanghai, China) was used to analyze the levels of NO production in MDMs, according to the manufacturer’s instructions. The concentration of nitrite was measured using absorbance readings at 540 nm. The levels of ATP were detected using the Enhanced ATP assay kit (Beyotime Biotechnology, Shanghai, China), according to the manufacturer’s instructions. The luminous value of ATP was read in Luminometer. Enhanced mitochondrial membrane potential assay kit (Beyotime Biotechnology, Shanghai, China) with JC-1 was used to analyze the levels of JC1 in MDMs. For detection with fluorescence microplate reader, excitation wavelength was 485 nm and emission wavelength was 590 nm. The activities of the mitochondrial complex I or IV were determined by the Mitochondrial Respiratory Chain Complex I or IV activity assay kit (Solarbio, Beijing, China) according to the manufacturer’s instructions. Briefly, mitochondrial homogenates were added into the respective reaction buffer. The reaction mixture was transferred to a microtiter plate and immediately put into a microplate reader (Thermo Fisher). The absorbance of reaction mixture was measured at 340 nm for Complex I and 550 nm for Complex IV, respectively. Mitochondrial complex activity was expressed as nmol/min/mg protein.

### Caspase-1, Caspase-3, and Caspase-8 Activity Assays

Caspase activity assay kits were used to detect the activity of caspases, according to the manufacturer’s instructions. Briefly, cell lysates were centrifuged at 13,000 g, 4°C, for 15 min, and the protein concentrations were determined by Bradford protein assay (Beyotime Biotechnology, Shanghai, China). Protein extracts were incubated with reagent from caspases activities kit in a 96-well microtiter plate at 37°C, for 2 h or overnight and detected at a wavelength of 405 nm using a MultiScan Go microplate reader (Thermo Fisher Scientific, Inc.).

### Western Blotting

Total proteins were extracted using IP cell lysate buffer with 1 mM PMSF. Proteins were resolved using SDS-polyacrylamide gel electrophoresis (4-15% SDS-PAGE) and transferred onto methanol-activated polyvinylidene difluoride (PVDF) membrane (Bio-Rad). The membranes were incubated with the aforementioned primary antibodies (1:1,000). Protein bands were visualized using a C600 ultrasensitive chemiluminescence imager, and expression levels were quantified using Image-Pro Plus software. The membrane-developed blots were developed, observed, and analyzed by using AlphaImager 2200 software (Alpha Innotech Corporation, CA, USA).

### Luciferase Reporter Gene Assay

The promoter region of *ACSL1* (−1 to −2,112 bp, −1 to −1,584bp, −1 to −1014 bp, −1 to −527 bp, −1 to −281 bp) was cloned into the pGL3-basic vector. DF-1 cells were plated at a density of 10^5^ cells per well in a 48-well plate. DF-1 cells were transiently transfected with 100 ng *ACSL1* promoter-luciferase reporter vectors and 5 ng Renilla luciferase reporter vectors plus Flag-*STAT1*, *IRF1* expressing plasmids using Lipofectamine 3000. After 24 h, cells were harvested and luciferase activities were measured with a dual-luciferase reporter assay system (Promega) according to the manufacturer’s instructions. Data are normalized for transfection efficiency by dividing Firefly luciferase activity by Renilla luciferase activity. Each experiment was replicated at least three times.

### Flow Cytometry

The prepared cells were added to 500 μl 1× Annexin V buffer, and the cells were gently resuspend. Cell suspensions were incubated with 5 μl Annexin V-FITC and 5 μl propidium iodide staining solution at 25°C for 15 min in the dark and then detected by flow cytometry.

### Confocal Microscopy

DF-1 cells were transfected with the expression plasmid for Flag-tagged ACSL1. After 24 h, cells were ALV-J infected or IFN-α treated for 16 h. The cells were fixed in 4% paraformaldehyde and permeabilized with 0.1% saponin, then blocked for 30 min with 10% skim milk, incubated overnight at 4°C with anti-Flag primary antibody. Cells were incubated with corresponding FITC-labeled secondary antibodies, and DAPI were used to stain the nucleus. Imaging of the cells was carried out using Leica TCS SP5 confocal laser microscopy under a ×40 objective.

### Statistical Analysis

Statistical comparisons were performed using GraphPad Prism (version 8.0) software (GraphPad Software Inc.). Student’s t test was used to analyze the data. Data shown are the means ± SEM. Differences with p < 0.05 were considered significant. *P < 0.05, **P < 0.01, ***P < 0.001.

## Results

### Promoter Region and Phylogenetic Analysis of *ACSL1*

To identify which nucleotides and regions are important in determining *ACSL1* transcription, the 2,112 bp nucleotide sequences upstream of the *ACSL1* coding region were truncated into five segments to construct different dual-luciferase reporter vectors. Luciferase reporter assays revealed that there were five abnormal fluctuations in *ACSL1* promoter region (**Supplementary Figure 1A**). Using the promoter prediction server (http://alggen.lsi.upc.es/), IRF1 and STAT1 as transcription factors were found in between −1,584 and −1,014 bp of *ACSL1* promoter region (**Supplementary Figure 1B**). Based on these analyses, we constructed *ACSL1* promoter reporter which contained the region between −1,584 and −1,014 bp (wt P3-P4-luc) and a mutated reporter (mt P3-P4-luc) construct with mutation of the IRF1 and STAT1 binding motifs. Then, *ACSL1* promoter reporter (wt P3-P4-luc) or its mutation (mt P3-P4-luc) were co-transfected with *IRF1* or *STAT1* expression plasmids into chicken embryo fibroblast cell lines (DF-1 cells). The luciferase reporter assays showed that when co-transfected with *IRF1* or *STAT1*, the fluorescence intensity value was increased in wt P3-P4-luc cells compared to mutation group (**Supplementary Figures 1C, D**). Thus, these findings suggest that IRF1 and STAT1 might be transcription factors, driving ACSL1 transcription in the IFN-I signaling pathway. Moreover, to identify the sequence characteristics of the chicken *ACSL1*, phylogenetic analyses of the relationship between *ACSL1* sequence of different species were performed, and the result showed that the chicken *ACSL1* most closely to the ring-necked pheasant and the turkey (**Supplementary Figure 1E**).

### ALV-J Infection Induces *ACSL1* Expression

**Figure 1:**
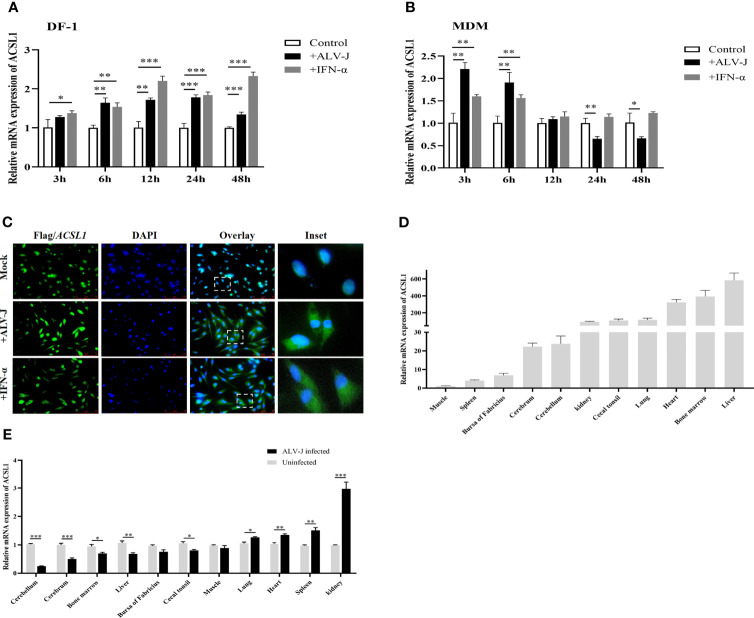
ALV-J infection induces *ACSL1* expression, an IFN stimulated gene. **(A, B)** DF-1 cells **(A)** and MDMs **(B)** were infected with ALV-J (10^4^ TCID_50_/0.1 ml) or treated with IFN-α (1,000 U/ml) for the indicated time. Relative mRNA levels of *ACSL1* were analyzed by quantitative RT-PCR (qRT-PCR). **(C)** Flag-*ACSL1* was expressed in DF-1 cells for 24 h, then the cells were infected with ALV-J (10^4^ TCID_50_/0.1 ml) or treated with IFN-α (1,000 U/ml). Immunofluorescence with anti-Flag antibody was used to detect the overexpressed protein (green). Nuclear was identified by DAPI (blue). Scale bar, 75 μm. Experiments were repeated at least three times independently, with similar results obtained. **(D)** Distribution of *ACSL1* in various specific-pathogen-free (SPF) White Leghorn chicken tissue following intraperitoneal injection of serum-free DMEM was determined using qRT-PCR analysis. **(E)** *ACSL1* mRNA expression change in various specific-pathogen-free (SPF) White Leghorn chicken tissue following intraperitoneal injection of ALV-J (0.2 ml, 10^4^ TCID_50_/0.1 ml) determined by qRT-PCR. Data shown are the means ± SEM (n=3). P values were calculated using two-tailed unpaired Student’ t-test. Differences with p < 0.05 were considered significant. *P < 0.05, **P < 0.01, ***P < 0.001.

To identify whether ACSL1 was an ISG, we examined the levels of ACSL1 in DF-1 cells or MDMs infected with ALV-J or stimulated with IFN-α. We found that ACSL1 was significantly induced upon ALV-J infection in DF-1 cells (**Figure 1A**) or IFN-α stimulation in MDMs (**Figure 1B**). It has been reported that ALV-J infection induces strong innate immune responses in MDMs at the early infection stage, instead of the late infection stage (13, 23). Expectedly, upon ALV-J infection, mRNA levels of ACSL1 in MDMs were significantly decreased by 24 h post-infection (hpi) (**Figure 1B**). Subsequently, we investigated the localization of ACSL1 in cells. We firstly overexpressed Flag-ACSL1 in DF-1 cells and then infected them with ALV-J (10^4^ TCID_50_/0.1 ml) or stimulated with alpha IFN (IFN-α, 1,000 U/ml). The result of immunofluorescence assay showed that ACSL1 was vividly located in the nucleus (**Figure 1C**). Interestingly, ACSL1 was observed in the cytoplasm and nucleus after being infected with ALV-J or treated with IFN-α (**Figure 1C**), suggesting that ALV-J or IFN-α regulates the localization of ACSL1. Moreover, the abundance of ACSL1 in chicken liver and bone marrow and the upregulation in immune-related tissues including kidney and spleen after intraperitoneal injection of ALV-J were also observed by quantitative RT-PCR (qRT-PCR) (**Figures 1D, E**). Collectively, ACSL1 is an ISG which could be upregulated upon ALV-J infection and IFN-α treatment.

### *ACSL1* Limits ALV-J Replication by Inducing IFN-Ⅰ Production

**Figure 2:**
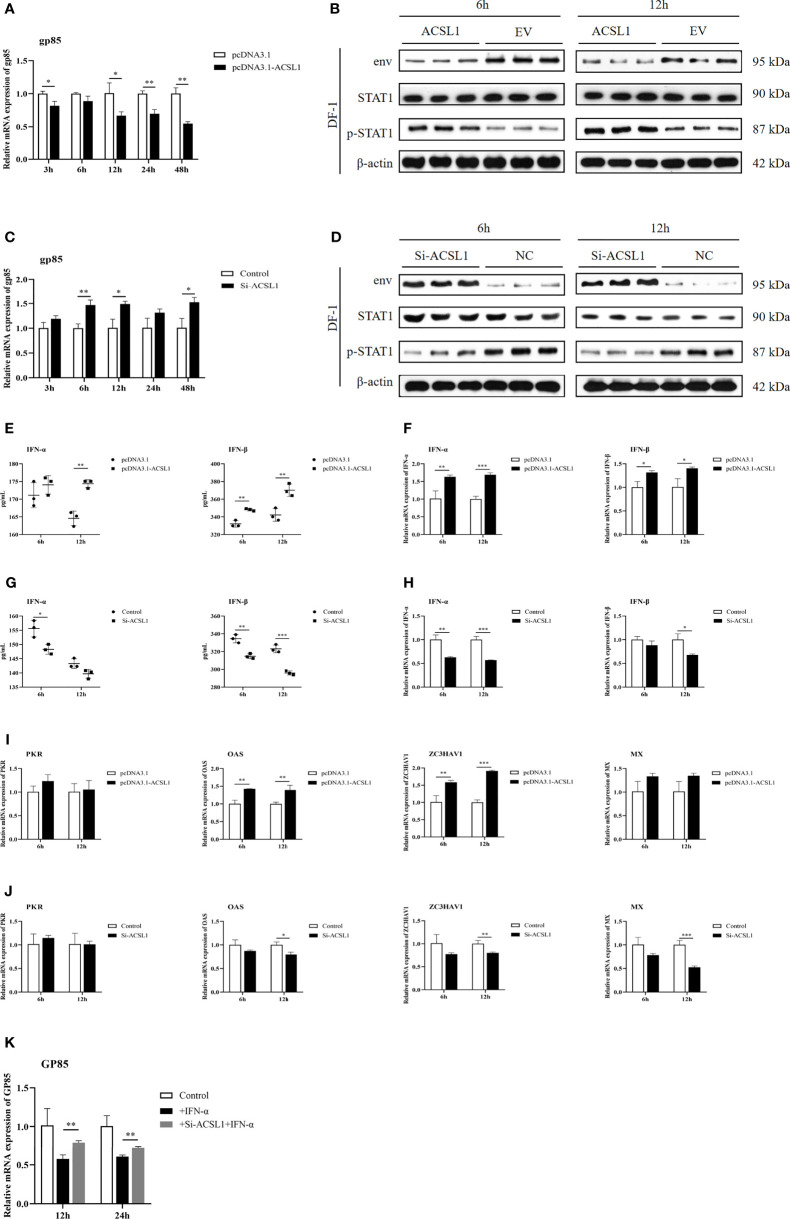
*ACSL1* inhibited ALV-J replication by enhancing IFN-I production. **(A, C)** *ACSL1* overexpression **(A)** and knockdown **(C)** in DF-1cells for 48 h and then infected with ALV-J (10^4^ TCID_50_/0.1 ml). Relative mRNA levels of gp85 were determined by qRT-PCR for the indicated time. **(B, D)** *ACSL1* overexpression and knockdown in DF-1 cells for 48 h and then infected with ALV-J (10^4^ TCID_50_/0.1 ml) for 6 and 12 h before assays. Immunoblot analysis of the levels of ALV-J envelope protein JE9 (env), STAT1, and p-STA1 from ACSL1 overexpression cells **(B)** or ACSL1 knockdown cells **(D)**. **(E, F)** The levels of IFN-α/IFN-β were determined by qRT-PCR **(E)** and ELISA **(F)** from ACSL1 overexpression cells. **(G, H)** qRT-PCR (G) and ELISA **(H)** analysis of the expression levels of IFN-α/IFN-β from ACSL1 knockdown cells. **(I, J)** qRT-PCR analysis of the expression levels of ISGs, including PKR, OAS, ZC3HAV1, and Mx from ACSL1 overexpression **(I)** or knockdown cells **(J)**. **(K)** DF-1 cells were transfected with small interfering RNA (siACSL1 or si-control) for 48 h prior to treatment with IFN-α (1,000 U/ml) for 1 h and then infected with ALV-J (10^4^ TCID_50_/0.1 ml). qRT-PCR analysis of gp85 level for the indicated time. Data shown are the means ± SEM (n=3). P values were calculated using two-tailed unpaired Student’ t-test. Differences with P < 0.05 were considered significant. *P < 0.05, **P < 0.01, ***P < 0.001.

To understand the function of ACSL1 during ALV-J infection, DF-1 cells were transfected with ACSL1 expression plasmid or small interfering RNA (siACSL1 or si-control) followed by ALV-J infection. The result of qRT-PCR and immunoblot analyses showed that the level of ALV-J was significantly decreased in cells overexpressing ACSL1 compared to empty vector control (**Figures 2A, B**), while the level of virus was greatly increased in cells transfected with siACSL1 (**Figures 2C, D**). Next, we sought to determine if ACSL1 suppresses ALV-J by affecting the level of IFN-I. Interestingly, we found that the levels of IFN-α/IFN-β in cells overexpressing ACSL1 were higher than that in control cells with ALV-J infection (**Figure 2E**). Consistently, the same results were confirmed in the qRT-PCR (**Figure 2F**). In contrast, when ACSL1 was knocked down in cells, the levels of IFN-α/IFN-β were both significantly decreased (**Figures 2G, H**). Meanwhile, mRNA expression of some ISGs, including OAS, ZC3HAV1, and Mx, was increased when ACSL1 was overexpressed in DF-1 cells, but reduced in the absence of ACSL1, whereas PKR was no significant difference (**Figures 2I, J**). Next, we found that the phosphorylation of STAT1 in cells overexpressing ACSL1 upon ALV-J infection was higher in comparison to the control cells (**Figure 2B**), suggesting that JAK/STAT signaling might be activated, which in turn enhances the expression of ISGs. Subsequently, we further evaluated the impact of ACSL1 on the antiviral efficiency of IFN-α against ALV-J. The result showed that IFN-α pretreatment reduced viral production in both groups; however, it was less efficient in protecting ACSL1 knockdown cells against ALV-J replication as compared to the control cells (**Figure 2K**). Taken together, our findings suggest that ACSL1 is involved in inhibiting ALV-J replication by positively regulating type IFN-I production.

### *ACSL1* Inhibits ALV-J Replication by Affecting the Activation of Akt

**Figure 3:**
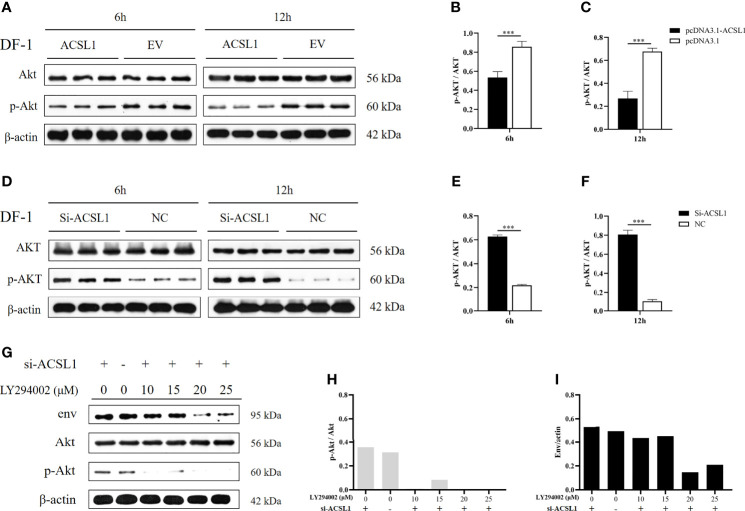
*ACSL1* inhibited the activation of Akt. **(A–C)** Immunoblot analysis of Akt and p-Akt expression from ACSL1 overexpression cells for the indicated time **(A)**. Statistical analysis of the relative abundance of protein between p-Akt and Akt **(B, C)**. **(D–F)** Immunoblot analysis of Akt and p-Akt expression from ACSL1 knockdown cells for the indicated time **(D)**. Statistical analysis of the relative abundance of protein between p-Akt and Akt **(E, F)**. **(G–I)** LY294002 were pretreated in *ACSL1* knockdown cells for 1h and then infected with ALV-J. Immunoblot analysis of env, Akt, and p-Akt expression for the 12 h **(G)**. **S**tatistical analysis of the relative abundance of protein between p-Akt and Akt **(H)**, env and actin **(I)**. The experiments were repeated three times, independently, with similar results obtained. Data shown are the means ± SEM (n=3). P values were calculated using two-tailed unpaired Student’ t-test. Differences with P < 0.05 were considered significant. ***P < 0.001.

A previous study found that ALV-J infection was discovered to activate the phosphorylation of Akt (25). We had identified ACSL1 as an inhibitor that might target the activation of Akt using the KEGG database (www.genome.jp/pathway/hsa04920+2180) (**Supplementary Figure 2**). Thus, we assumed that ACSL1 inhibits ALV-J replication by affecting the activation of Akt. To verify this hypothesis, we first overexpressed ACSL1 in DF-1 cells prior to infection with ALV-J and then performed immunoblot assays. As expected, the phosphorylation of Akt in overexpressed ACSL1 cells was significantly decreased (**Figures 3A–C**). In contrast, knockdown of ACSL1 increased the phosphorylation of Akt (**Figures 3D–F**). Next, a phosphatidylinositol 3-kinase (PI3K) inhibitor, LY294002, was pretreated in *ACSL1* knockdown cells for 1 h and then infected with ALV-J (**Figure 3G**). The result showed that the level of ALV-J was significantly decreased in *ACSL1* knockdown cells with LY294002 pretreatment (**Figures 3G–I**). Collectively, these data suggest that *ACSL1* inhibits ALV-J replication through decreasing the phosphorylation of Akt.

### *ACSL1* Induces Apoptosis Through PI3K/Akt Signaling Pathway in MDMs During ALV-J Infection

**Figure 4:**
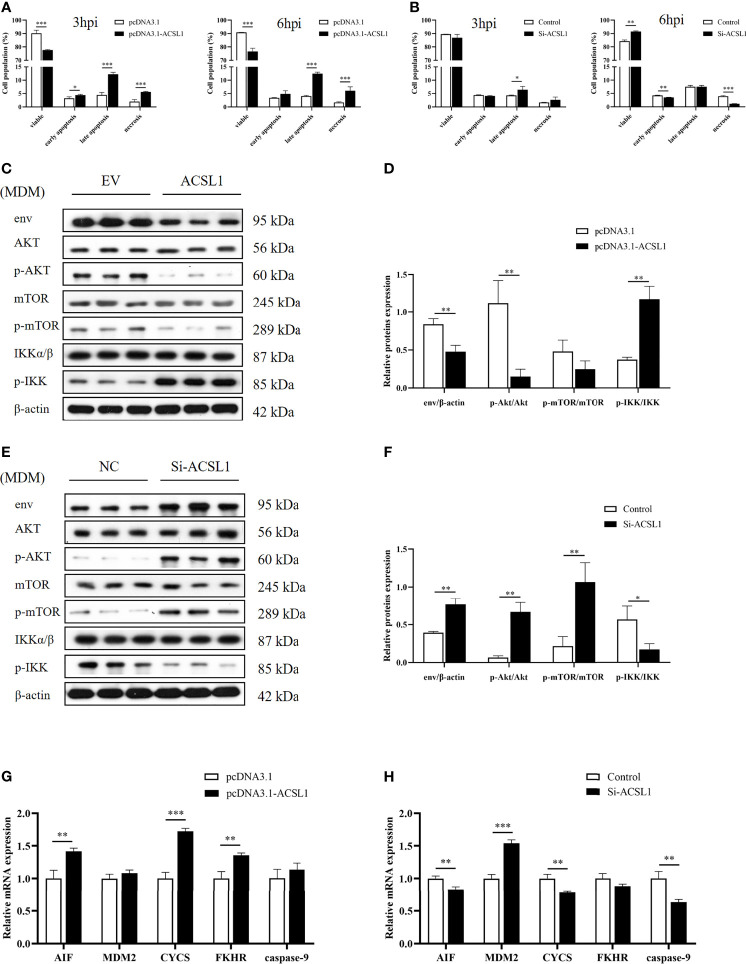
*ACSL1* induced apoptosis *via* PI3K/Akt signaling pathway. **(A, B)** *ACSL1* overexpression **(A)** and knockdown **(B)** in MDMs for 48 h, followed by infection with ALV-J (10^4^ TCID_50_/0.1 ml) before assays. Apoptosis analysis of MDMs for 3 and 6 hpi, using Annexin V-FITC. Statistical analysis of the data from the multiple repeated Annexin V-FITC experiments. **(C, D)** Immunoblot analysis of the levels of env, Akt, p-Akt, mTOR, p-mTOR, IKKα/β, and p-IKK expression in MDMs transfected with *ACSL1* expression plasmids or empty vector control for 48 h followed by ALV-J infection for 3 h. **(E, F)** Immunoblot analysis of the levels of env, Akt, p-Akt, mTOR, p-mTOR, IKKα/β, and p-IKK expression in MDMs transfected with siACSL1 or control siRNA for 48 h followed by ALV-J infection for 3 h**. (G, H)** qRT-PCR analysis of apoptosis-related genes (*AIF*, *CYCS*, *FKHR*, *MDM2*, and *caspase-9*) in *ACSL1* overexpressed cells **(G)** or *ACSL1* knockdown cells **(H)** followed by ALV-J infection for 3 h. Data shown are the means ± SEM (n=3). P values were calculated using two-tailed unpaired Student’s t-test. Differences with P < 0.05 were considered significant. *P < 0.05, **P < 0.01, ***P < 0.001.

Phosphatidylinositol 3-kinase (PI3K)/protein kinase B (PKB/Akt) signaling pathway is responsible for regulating cell survival (26). A previous study found that ALV-J infection can induce chicken monocyte apoptosis (27). Thus, we hypothesized that *ACSL1* induces apoptosis *via* the PI3K/Akt signaling pathway, which in turn affects ALV-J replication. To test this hypothesis, we performed flow cytometry analysis of apoptosis in MDMs which were overexpressed or knockdown ACSL1 followed by infecting with ALV-J. The result showed that the apoptosis was significantly increased in MDMs overexpressing ACSL1 compared to empty vector control (**Figure 4A** and **Supplementary Figures 3A, B**). In contrast, when siACSL1 was transfected in MDMs, the apoptosis was significantly decreased (**Figure 4B** and **Supplementary Figures 3C, D**). Subsequently, the expression levels of apoptosis-related genes in the PI3K/Akt signaling pathway were analyzed using immunoblot and qRT-PCR. Compared with the control cells, the levels of phosphorylated Akt were markedly decreased, while the levels of phosphorylated IKK were increased in MDMs overexpressing ACSL1 (**Figures 4C, D**). When in ACSL1 knockdown cells, the results have been reversed verified (**Figures 4E, F**). Moreover, the mRNA expression of these relative genes was significantly upregulated in MDMs overexpressing ACSL1 compared to empty vector control, including *AIF*, *CYCS*, and *FKHR*, while *caspase-9* was not significantly different (**Figure 4G**). Similarly, the opposite results have been verified in ACSL1 knockdown cells (**Figure 4H**). Overall, the above evidence supports that ACSL1 inhibited ALV-J replication *via* the PI3K/Akt signaling pathway–induced apoptosis.

### *ACSL1* Enhances MDMs-Dependent Inflammation During ALV-J Infection

**Figure 5:**
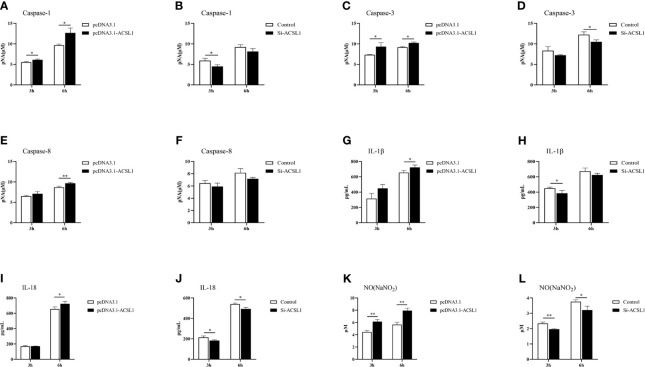
*ACSL1* promoted inflammation in MDMs. **(A–F)** Caspase-1, caspase-3, and caspase-8 activity assay kits were used to analyze caspase-1 **(A, B)**, caspase-3 **(C, D)**, and caspase-8 **(E, F)** enzyme activities in *ACSL1* overexpressed cells or *ACSL1* knockdown cells followed by ALV-J infection for 3 and 6 h. **(G–J)** The supernatants were harvested to examine the levels of IL-1β **(G, H)** and IL-18 **(I, J)** by ELISA. **(K, L)** The supernatants were harvested to examine the concentration of NO by NO commercial kit. Data shown are the means ± SEM (n=3). P values were calculated using two-tailed unpaired Student’s t-test. Differences with P < 0.05 were considered significant. *P < 0.05, **P < 0.01.

Since *ACSL1* promoted apoptosis and the levels of phosphorylated mTOR and IKK were affected, we hypothesized that ACSL1 may alter the expression of inflammatory cytokines during ALV-J infection. Caspase-1, caspase-3, and caspase-8 are important executors that mediate apoptosis and inflammation (28–30). Therefore, we examined the protein activity of caspase-1, caspase-3, and caspase-8 in MDMs with ACSL1 overexpression or knockdown during ALV-J infection. The results showed that caspase-1, caspase-3, and caspase-8 activities were significantly increased in ACSL1 overexpressed cells compared to control cells (**Figures 5A, C, E**). Conversely, knockdown of ACSL1 inhibited their activities (**Figures 5B, D, F**). The qRT-PCR results of the expression levels of caspase-1, caspase-3, and caspase-8 were increased in ACSL1 overexpressed cells, while decreased in ACSL1 knockdown cells (**Supplementary Figures 4A–F**).

Considering activated caspase-1 can mediate proteolytic cleavage of the pro-inflammatory precursor cytokines pro-interleukin-1β (IL-1β) and pro-IL-18 (31, 32), we further examined the levels of IL-1β and IL-18 in MDMs with ACSL1 overexpression or knockdown upon ALV-J infection. The results of qRT-PCR and ELISA analyses showed that the levels of IL-1β and IL-18 were significantly increased in ACSL1 overexpressed cells, while were decreased in ACSL1 knockdown cells (**Figures 5G–J** and **Supplementary Figures 4G–J**). Moreover, we found that the levels of nitric oxide (NO) and inducible nitric oxide synthase (iNOS) in overexpressed ACSL1 cells were remarkably increased. Conversely, the opposite results have been verified in ACSL1 knockdown cells (**Figures 5K, L** and **Supplementary Figures 4K, L**). Together, these findings prove that ACSL1 may enhance inflammation to fight against ALV-J infection in MDMs.

### ACSL1 Induces MDMs to Pro-Inflammatory Phenotypic States During ALV-J Infection

**Figure 6:**
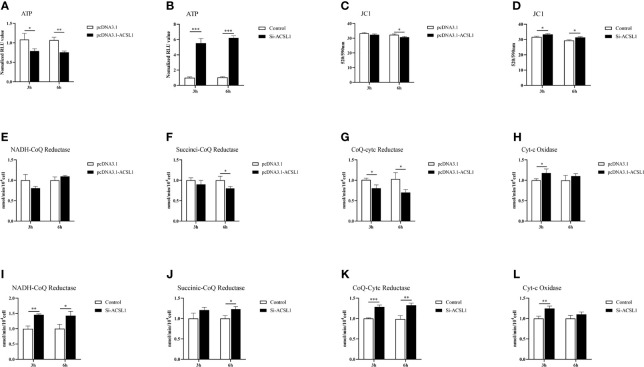
ACSL1 induces MDMs to pro-inflammatory phenotypic states. **(A, B)** The levels of ATP were detected by Enhanced ATP assay kit. **(C–D)** The levels of mitochondrial membrane potential JC1 were detected by Enhanced mitochondrial membrane potential assay kit with JC-1. **(E–L)** Mitochondrial respiratory chain complexes activity detection kit was used to analyze mitochondrial respiratory chain enzyme activities. Data shown are the means ± SEM (n=3). P values were calculated using two-tailed unpaired Student’s t-test. Differences with P < 0.05 were considered significant. *P < 0.05, **P < 0.01, ***P < 0.001.

It has long been known that macrophages can be driven to specific states of activation following exposure to defined environmental cues and cytokines (33). So, we suspected that ACSL1 would change the functional states of MDMs for inhibiting ALV-J replication. We have confirmed ACSL1 enhanced the antiviral functions of MDMs, including upregulation of the levels of IL-1β and IL-18, and induced the production of NO (**Figures 5G–L**). Next, we examined the levels of triphosphate (ATP) and mitochondrial membrane potential JC1. The results showed that upon infection, the levels of ATP and JC1 were decreased in MDMs overexpressing ACSL1, while were increased in ACSL1 knockdown cells (**Figures 6A–D**). The levels of JC1 were decreased, meaning there was occurrence of early apoptosis in MDMs.

Due to changes in the levels of ATP, we speculate that the activity of mitochondrial respiratory chain enzymes will also change accordingly. The activities of mitochondrial respiratory chain complexes I, II, and III were all reduced to varying degrees in MDMs overexpressing ACSL1 (**Figures 6E–H**). When in ACSL1 knockdown cells, the activities of mitochondrial respiratory chain complexes all showed increased (**Figures 6I–L**). These results suggest that ACSL1 induces MDMs to pro-inflammatory phenotypic states, which in turn enhances the antiviral response.

## Discussion

**Figure 7:**
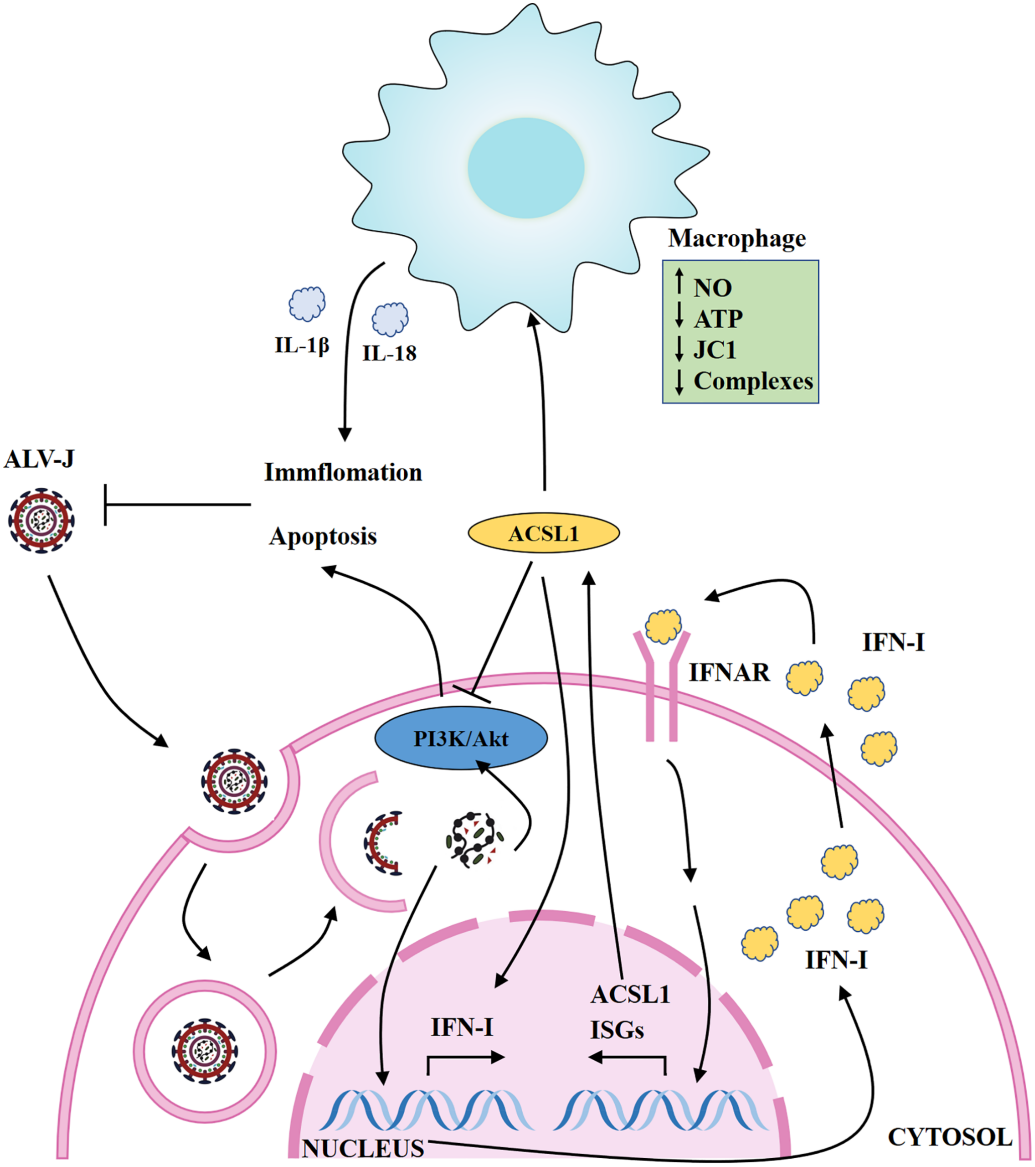
Schematic map of ACSL1 regulating ALV-J replication. ACSL1 positively regulates type IFN-I production, induces apoptosis *via* the PI3K/Akt signaling pathway, and promotes inflammation, which in turn inhibits ALV-J replication. IFNAR, interferon receptors; ISGs, interferon stimulate genes.

The main reason for the difficulty in vaccine or drugs development of ALV-J is the genetic instability and variability of the retrovirus. Severe economic losses caused by ALV-J remain an unsolved problem in the world due to inefficient eradication strategies and lack of effective vaccines (34). IFN-I is the important element in provoking the host innate immunity system against viruses. Although ALV-J can be sensitive to IFN-I-induced inhibition (12), the specific ISGs that mediate the anti-ALV-J activity of IFNs have not yet been fully defined. Here, we have demonstrated that one ISG, ACSL1, can restrict the replication of ALV-J by positively regulating type IFN-I production, and induce apoptosis *via* the PI3K/Akt signaling pathway. In addition, ACSL1 activates MDMs to pro-inflammatory phenotypic states during ALV-J infection (**Figure 7**).

In this study, we found that ACSL1 has various function during ALV-J infection. We observed that ACSL1 inhibiting ALV-J replication is largely due to the higher IFN-I production. IFNs induce activation of the JAK/STAT signaling pathway, which in turn stimulate the transcription of ISGs (35). As expected, the phosphorylation of STAT1 and some ISGs was significantly increased after ACSL1 overexpression. Therefore, it suggested that the relationship between ACSL1 and IFN may be positive feedback regulated. Unfortunately, the detailed mechanism of how *ACSL1* promotes IFN-I is unknown in this study.

Specifically, upon ALV-J infection, ACSL1 promotes apoptosis *via* the PI3K/Akt signaling pathway and induces an increase in inflammation. Viruses regulating the activation of signaling pathway is one of the effective means to promote their survival or aid their infection at different stages of the viral life cycle (36). Activation of the PI3K/Akt signaling pathway is one of the early infection events of ALV-J (25), while PI3K/Akt signaling pathway plays an important role in proliferation and apoptosis (37). After the detection of the levels of apoptosis-related genes, we found that ACSL1 triggers apoptosis through the PI3K/Akt signaling pathway. Likewise, upon ALV-J infection, we also observed that the activation of apoptosis-related proteins caspase-1, caspase-3, and caspase-8 is increased in ACSL1-overexpressed cells. The activation of caspase-1 leads to the release of inflammatory hallmarks IL-1β and IL-18 (28). As anticipated, we found that ACSL1 overexpression elevates levels of pro-inflammatory cytokines, including IL-1β and IL-18. IL-1β is a potent inducer of inflammation, vasodilation, and immune cell extravasation (38). IL-18 promotes IFN-γ production in TH1 cells, NK cells, and cytotoxic T cells, enhancing the development of TH2 cells and promoting local inflammation (39). Moreover, IL-1β and IL-18 as pro-inflammatory cytokines prevent the infection of pathogens effectively, yet excess pro-inflammatory cytokines is harmful to the host. Importantly, although apoptosis has generally been considered an immunologically silent process, emerging evidence indicates that apoptosis can be inflammatory when induced under certain conditions and has roles in the host defense against infection (40–42). In the present study, our results prove the above point of view. The *in vivo* reality is of much greater nuance and complexity, so further research on this aspect is needed *in vivo*.

In addition to the abovementioned data, we were pleasantly surprised to find that ACSL1 induces MDMs to pro-inflammatory phenotypic states during ALV-J infection. Similar to M1-like macrophages polarization states (43), *ACSL1* overexpressing MDMs show enhanced microbicidal responses, including production of inflammatory, generation of NO, change of ATP, JC1, and mitochondrial respiratory chain complexes. Currently, there are relatively few descriptions about ISG changes in the phenotypic or metabolic function of macrophages, thus whether and how ISGs shape macrophage polarization that affect pathogen infection remains an open question.

In summary, we have identified a novel ISG, ACSL1, that is important to inhibit ALV-J replication. Inhibition of virus replication in a host population can significantly reduce the change of viral adaption (44). The availability of genome-editing tools, notably CRISPR/Cas9, widens the scope of animal breeding and its applications in the context of disease control, while targeted genome editing has been proven useful to resistant ALV-J in chickens (34, 44, 45). In this context, exploiting more antiviral genes are particularly valuable in chickens. Thus, our work not only polishes our knowledge about the immune response strategies between the host and ALV-J but also provides valuable targets for gene-engineering or treating ALV-J infection-triggered diseases.

## Data Availability Statement

The original contributions presented in the study are included in the article/**Supplementary Material**. Further inquiries can be directed to the corresponding author.

## Ethics Statement

The animal study was reviewed and approved by South China Agricultural University. Written informed consent was obtained from the owners for the participation of their animals in this study.

## Author Contributions

QZ conceived and performed the experiments, analyzed the data, and wrote the manuscript. TX and GM conceived the experiments and revised the manuscript. ZZ and LL analyzed data and assisted in the cell culture work. XZ revised and approved the final manuscript. All authors contributed to the article and approved the submitted version.

## Funding

This work was supported by the National Natural Science Foundation of China (grant numbers 31801030 and 31571269) and the China Agriculture Research System (grant number CARS-41-G03).

## Conflict of Interest

The authors declare that the research was conducted in the absence of any commercial or financial relationships that could be construed as a potential conflict of interest.

## Publisher’s Note

All claims expressed in this article are solely those of the authors and do not necessarily represent those of their affiliated organizations, or those of the publisher, the editors and the reviewers. Any product that may be evaluated in this article, or claim that may be made by its manufacturer, is not guaranteed or endorsed by the publisher.
