# Supplementary material for: ACSL1 Inhibits ALV-J Replication by IFN-Ⅰ Signaling and PI3K/Akt Pathway
Source: Front Immunol. 2021 Oct 29;12:774323. doi: 10.3389/fimmu.2021.774323 (PMC8585972; doi:10.3389/fimmu.2021.774323)
Supplement: Supplementary file 1 [file DataSheet_1.docx]

**SUPPLEMENTARY MATERIALS**

**TABLE**

**Table 1**

The primers used for the quantitative real-time PCR.

| Primer^a^ | Sequence (5'–3') | Accession no. |
| --- | --- | --- |
| *gp85-F* | TGTGTGCGTGGTTATTATTTC | DQ316906 |
| *gp85-R* | AATGGCGAGGTCGCTGACTGC |  |
| *iNOS-F* | TGCATTCAAACCTCATGCTG | NM_204961.1 |
| *iNOS-R* | CTGTGTAATGTGGACTGTCG |  |
| *ACSL1-F* | TTTGGACAGGCGGATACATCA | NM_001012578.1 |
| *ACSL1-R* | CCTCCCAAACTTGCCTGTATTTT |  |
| *AIF-F* | ATGTCGTGCTGCCGCCTG | NM_001007490.1 |
| *AIF-R* | CTCCTTGTCAGTGACCCCGT |  |
| *Caspase-1-F* | GAGTAATGGGACCACGGACA | AF031351.1 |
| *Caspase-1-R* | ACACGTCTATGCACTGAGGTA |  |
| *Caspase-3-F* | AAGATGGACCACGCTCAGGG | NM_204725.1 |
| *Caspase-3-R* | AGGTGGCTCTATGGCCTGAC |  |
| *Caspase-8-F* | CTGAAGTTTCTCAGCCTGGA | NM_204592.2 |
| *Caspase-8-R* | GTCCAAGGTCCGTCCCGTGC |  |
| *Caspase-9-F* | ACCGGCACCCGAAGGAGCAA | AY057940.1 |
| *Caspase-9-R* | CTTGCGAACAGGTAGGGTCA |  |
| *CYCS-F* | GGGAGATATTGAGAAGGGCAAG | NM_001079478.1 |
| *CYCS-R* | TGAACCCCACTCCTATGAGA |  |
| *FKHR-F* | TCGATCCGTCACAACTTCTCCC | AF114261.1 |
| *FKHR-R* | GGGTCGGAGACTGTCATTACTAC |  |
| *GAPDH-F* | GAACATCATCCCAGCGTCCA | NM_204305.1 |
| *GAPDH-R* | CGGCAGGTCAGGTCAACAAC |  |
| *IFN-α-F* | CAGGATGCCACCTTCTCTCAC | NM_205427.1 |
| *IFN-α-R* | AGGATGGTGTCGTTGAAGGAG |  |
| *IFN-β-F* | GCCCACACACTCCAAAACACTG | NM 001024836.1 |
| *IFN-β-R* | TTGATGCTGAGGTGAGCGTTG |  |
| *IL-1β-F* | ATGGCGTTCGTTCCCGACCTG | NM_204524.1 |
| *IL-1β-R* | CGGTACTGGTTTGACGACGCC |  |
| *IL-18-F* | ATGAGCTGTGAAGAGATCGC | GU119895.1 |
| *IL-18-R* | CTCCACTTTAGACCGTCACCT |  |
| *MDM2-F* | ATGTGCAATACCGAGATGACCT | NM_001199384.1 |
| *MDM2-R* | GACACGTTTACTAGAAGAAGAACCC |  |
| *MX-F* | TTGTCTGGTGTTGCTCTTCCT | NM_204609.1 |
| *MX-R* | GCTGTATTTCTGTGTTGCGGTA |  |
| *OSA-F* | CACGGCCTCTTCTACGACA | NM_205041.1 |
| *OSA-R* | TGGGCCATACGGTGTAGACT |  |
| *PKR-F* | CCTCTGCTGGCCTTACTGTCA | NM_204487.1 |
| *PKR-R* | AAGAGAGGCAGAAGGAATAATTTGCC |  |
| *ZC3HAV1-F* | TTGATTCGGCGCCTCTCTAC | NM_001012938.1 |
| *ZC3HAV1-R* | ACTGGCCGTGGTCATTCTTC |  |


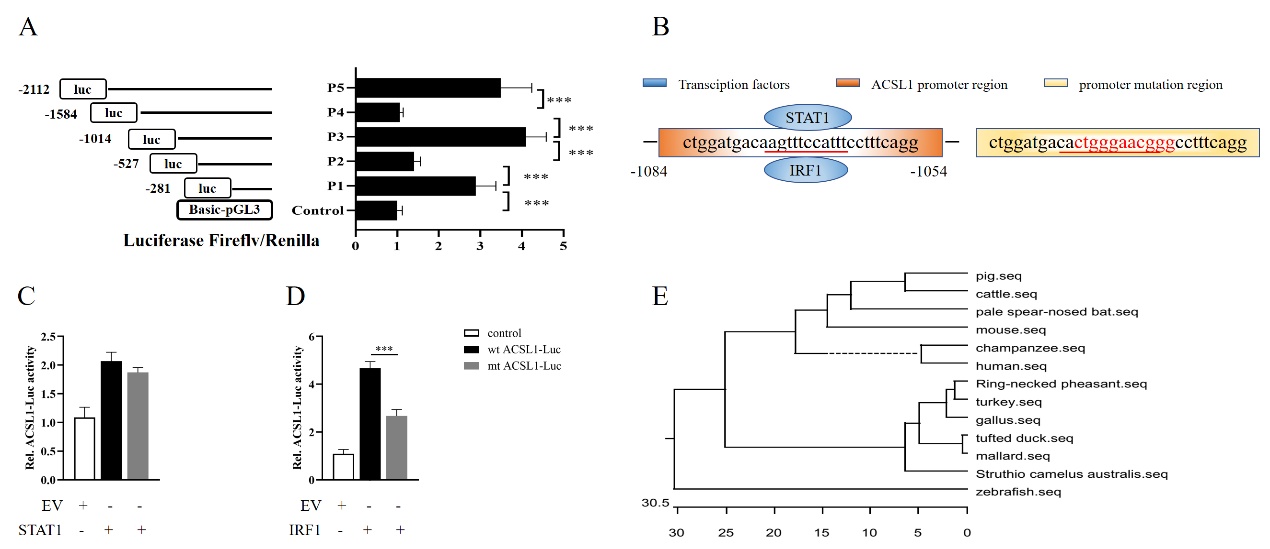


**Supplementary Fig. 1 Promoter region and phylogenetic analysis of *ACSL1*.**

**(A),** DF-1 cells were transfected with a series of dual luciferase reporter vectors containing the promoter of the *ACSL1* (luc-2112, luc-1584, luc-1014, luc-527, luc-281, and pGL3-basic), then dual luciferase reporter assay was performed after 48 h.

**(B),** Schematic representation of *ACSL1* promoter region bound by STAT1 and IRF1.

**(C, D),** Luciferase activities determined form DF-1 cells transfected with *ACSL1* promoter (wt P3-P4-luc) or its mutation (mt P3-P4-luc) together with *STAT1*, *IRF1* expressing plasmids.

**(E).** A maximum-likelihood tree was generated using 100 bootstrap values to show the similarity of orthologous *ACSL1*s.

Data shown are the means ± SEM (n=3). P values were calculated using two-tailed unpaired Student’ t-test. Differences with p < 0.05 were considered significant. *P < 0.05, **P < 0.01, ***P < 0.001.


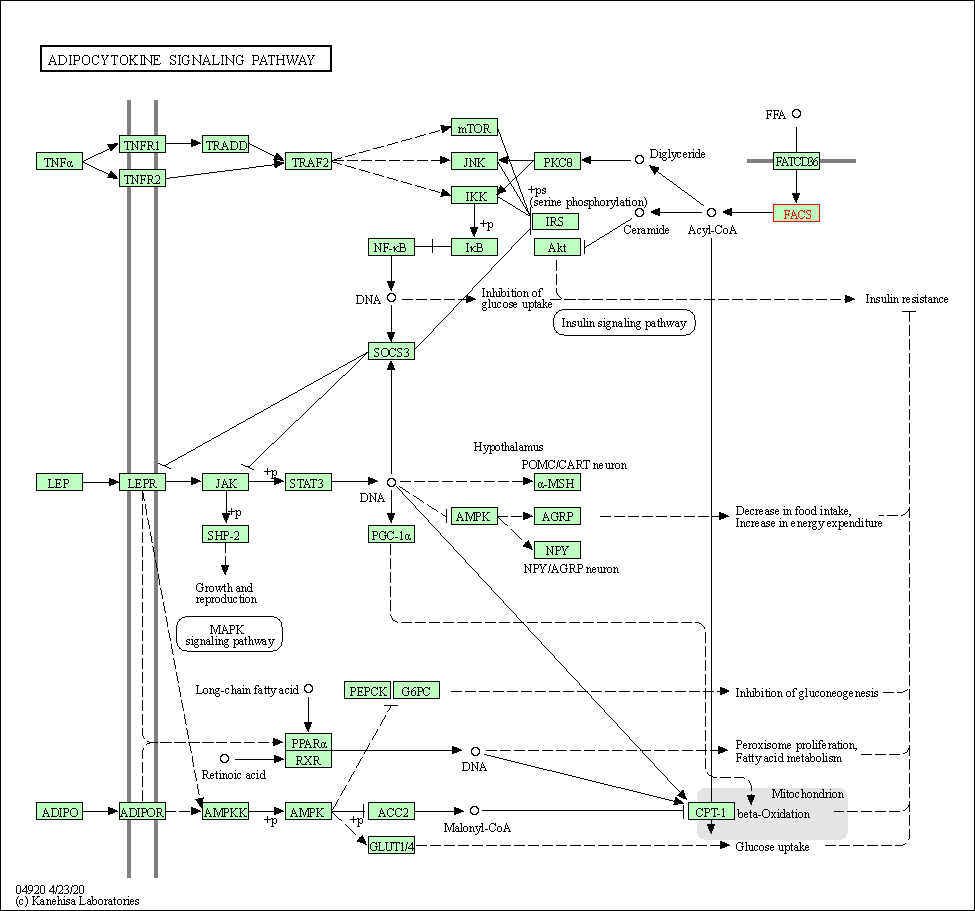


**Supplementary Fig. 2 *ACSL1* inhibited the activation of Akt.**

Schematic pathway of the relationship between *ACSL1* (FACS) and Akt. The image from KEGG database ([www.genome.jp/pathway/hsa04920+2180](http://www.genome.jp/pathway/hsa04920+2180)).


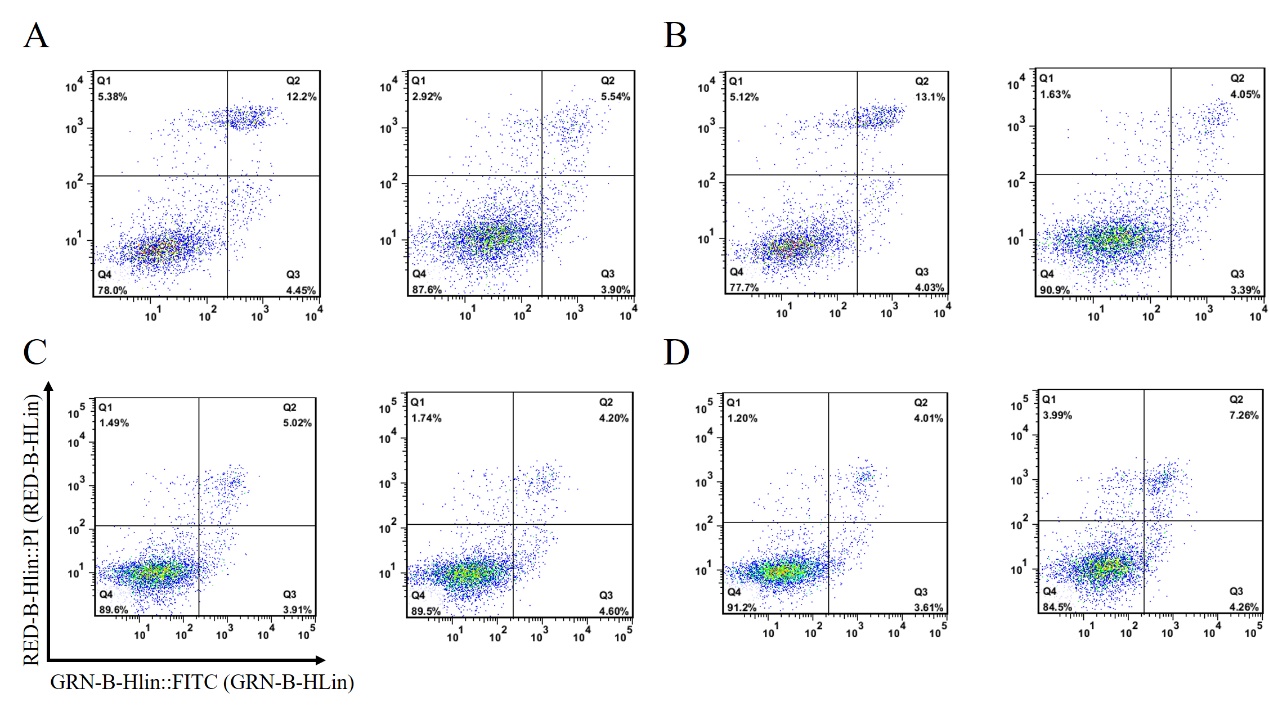


**Supplementary Fig. 3 *ACSL1* induced apoptosis in MDMs.**

**(A-D),** *ACSL1* overexpression **(A, B)** and knockdown **(C-D)** in MDMs for 48 h, followed by infection with ALV-J (10^4^ TCID_50_/0.1 mL) for 3 h (**A, C**), 6 h (**B, D**). Apoptosis were analyzed by flow cytometry.

The experiments were repeated three times, independently, with one of the results shown. Q1 is the percentage of necrotic cells or cell debris. Q2 is the percentage of cells with advanced apoptosis. Q3 is the percentage of cells in early apoptosis. Q4 is the percentage of viable cells.


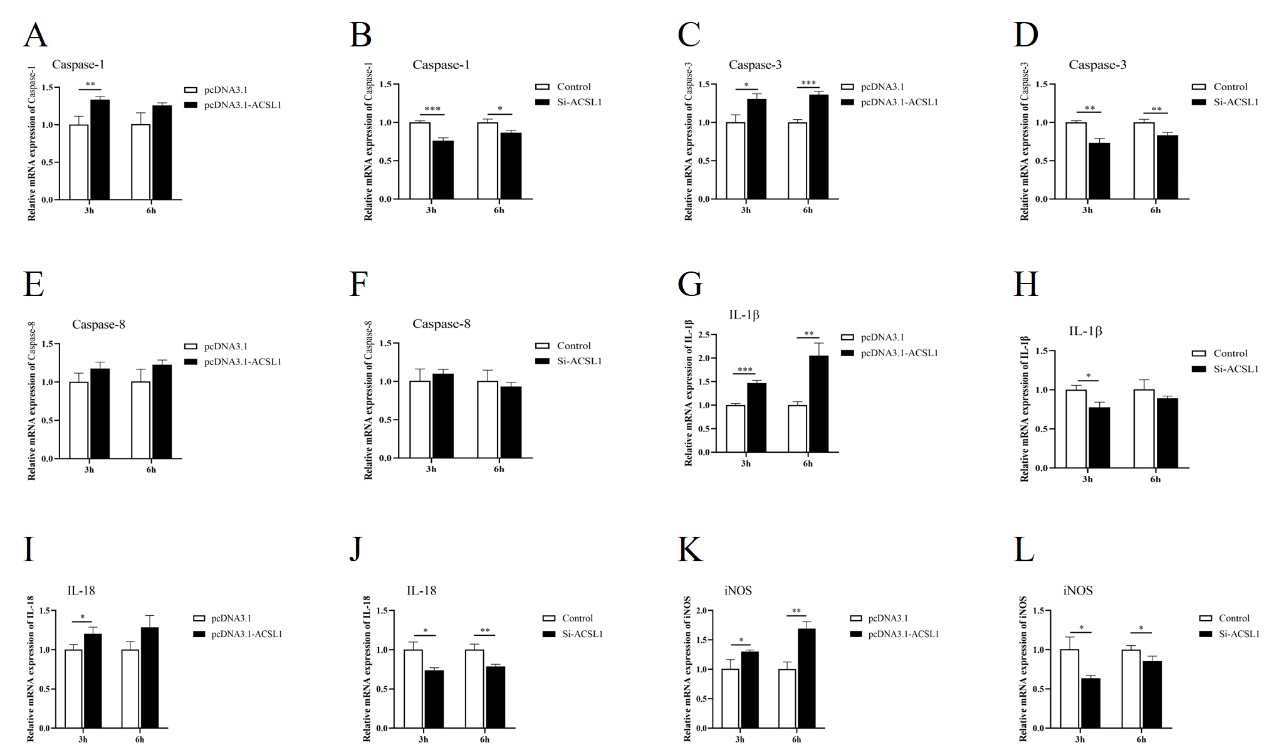


**Supplementary Fig. 4 *ACSL1* promoted inflammation in MDMs.**

**(A-L),** *ACSL1* overexpression and knockdown in MDMs for 48 h, followed by infection with ALV-J (10^4^ TCID_50_/0.1 mL) for 3, 6 h before assays. qRT-PCR analysis of the levels of caspase-1 **(A, B)**, caspase-3 **(C, D)**, caspase-8 **(E, F)**, IL-1β **(G, H)**, IL-18 **(I, J)**, and iNOS **(K, L)**.

Data shown are the means ± SEM (n=3). P values were calculated using two-tailed unpaired Student’ t-test. Differences with P < 0.05 were considered significant. *P < 0.05, **P < 0.01, ***P < 0.001.
